# Genetic polymorphisms of antioxidant enzymes (GSTP1/CAT/HMOX1/EPHX1) and childhood asthma risk in Fuzhou

**DOI:** 10.3389/fped.2025.1524055

**Published:** 2025-05-09

**Authors:** Ziling Wu, Qiaobin Chen, CaiChun Lin, HongBiao Huang, Lang Chen

**Affiliations:** ^1^ Provincial Clinical Medical College of Fujian Medical University, Fujian Provincial Hospital, Fuzhou University Affiliated Provincial Hospital, Fuzhou, China; ^2^ Ningde People's Hospital, Ningde, China

**Keywords:** bronchial asthma, single nucleotide polymorphisms, antioxidant enzymes, GSTP1, CAT, HMOX1, EPHX1

## Abstract

**Objective:**

Discuss the correlation between single nucleotide polymorphisms (SNPs) of the Glutathione s-transferase Pi-1 (GSTP1), Catalase (CAT), Heme oxygenase-1 (HMOX1), and Homo sapiens epoxide hydrolase 1 (EPHX1) genes and the risk of childhood asthma in Fuzhou.

**Methods:**

Next generation sequencing (*N*GS) was employed to conduct whole-exome sequencing (WES) on 50 asthmatic children and 50 healthy children. Genetic models for the GSTP1 gene rs1695, rs4891, HMOX1 gene rs2071747, rs17878790, CAT gene rs7943316, rs1049982, rs769217, and EPHX1 gene rs2234922, rs41266231, rs1051740 sites were constructed. Binary logistic regression, linkage disequilibrium analysis, haplotype analysis, and interaction analysis were used to study the correlation between the 10 SNPs of GSTP1, CAT, HMOX1, and EPHX1 genes and the risk of asthma in children in the Fuzhou region.

**Results:**

The rs1695 A>G variant increased the risk of asthma in the heterozygous, dominant, and allele models. The rs4891 T>C variant increased the risk of asthma in the heterozygous, dominant, and allele models. The rs7943316 A>T variant increased the risk of asthma in the homozygous, recessive, and allele models. The rs769217 C>T variant decreased the risk of asthma in the homozygous, recessive, and allele models. Strong linkage disequilibrium between the GSTP1 gene rs1695 and rs4891, and the CAT gene rs7943316, rs1049982, and rs769217. The GC haplotype composed of GSTP1 gene rs1695 and rs4891 may pose a risk for childhood asthma [*P* = 0.025, OR = 2.12 (1.09–4.10)], while the AT haplotype may be protective [*P* = 0.025, OR = 0.47 (0.24–0.92)]. The ATT haplotype composed of CAT gene rs7943316, rs1049982, and rs769217 may be protective against childhood asthma [*P* = 0.006, OR = 0.45 (0.25–0.79)]. Potential synergistic interaction between the GSTP1 gene rs1695, CAT gene rs7943316, and EPHX1 gene rs41266231. The combination of GSTP1 gene rs1695 and CAT gene rs7943316 formed the best predictive model for assessing the risk of childhood asthma in the Fuzhou region.

**Conclusion:**

The genotype GC, composed of GSTP1 gene rs1695 and rs4891, may represent a risk genotype for childhood asthma, whereas genotype AT may represent a protective genotype for childhood asthma. The genotype ATT, composed of CAT gene rs7943316, rs1049982, and rs769217, may represent a protective genotype for childhood asthma. The combination of GSTP1 gene rs1695 and CAT gene rs7943316 constitutes the optimal model for predicting the risk of childhood asthma in the Fuzhou region.

## 1 Introduction

Bronchial asthma is one of the most common chronic non-communicable diseases globally (1), The onset of asthma is influenced by various factors including genetics, oxidative stress damage, allergen exposure, viral infections, environmental factors, and microbial exposure (2–4). Among these factors, genetics play a crucial role, with heritability estimates for asthma possibly reaching 60%–70% (5). Previous genome-wide association studies (GWAS) have identified numerous single nucleotide polymorphisms (SNPs) in genes associated with the risk of asthma (6), such as orosomucoid 1-like protein 3 (ORMDL3) gene rs7216389 (7), interleukin-6 (IL-6) gene rs1800795 (8), and thymic stromal lymphopoietin (TSLP) gene rs1837253 (9). These gene SNPs alter disease susceptibility by affecting protein synthesis levels encoded by the genes and the duration of protein action.

Oxidative stress damage plays a crucial role in the pathogenesis of asthma. Current research indicates that oxidative stress (OS) is one of the main pathophysiological mechanisms of chronic respiratory diseases. Oxidative stress is closely linked to asthma (10, 11) and contributes to asthma pathogenesis and progression by increasing infiltration of inflammatory cells in the airways, secretion of inflammatory mediators, promoting airway remodeling, increasing airway hyperresponsiveness, and reducing responsiveness of airway epithelial cells to corticosteroids (12). Oxidative stress in asthma is primarily driven by the dysregulation of key antioxidant enzymes. GSTP1 plays a critical role in detoxifying reactive oxygen species (ROS) by catalyzing the conjugation of glutathione to electrophilic compounds. The rs1695 (Ile105Val) polymorphism results in an amino acid substitution within the hydrophobic substrate-binding domain of enzyme, impairing its catalytic activity and exacerbating oxidative damage (13). Similarly, the CAT rs7943316 variant diminishes catalase activity, leading to the accumulation of hydrogen peroxide (H_2_O_2_) in bronchial epithelial cells and potentiating NF-*κ*B-mediated proinflammatory signaling. HMOX1 mitigates oxidative stress by degrading pro-oxidant heme into antioxidant byproducts. However, genetic variants such as rs17878790 may compromise its anti-inflammatory properties. Additionally, EPHX1 metabolizes environmental epoxide derivatives, may indirectly contribute to oxidative stress when functionally impaired. Collectively, these genes constitute an integrated regulatory network that maintains airway redox homeostasis, and their polymorphic interactions may synergistically modulate asthma pathogenesis (13–16).

It is no clear conclusion on whether these SNPs in antioxidant enzyme genes affect the risk of childhood asthma. To date, there is no research on the association between GSTP1, CAT, HMOX1, EPHX1 gene SNPs and the risk of childhood asthma in the Fuzhou region. This study preliminarily explores the correlation between GSTP1 gene rs1695, rs4891 sites; HMOX1 gene rs2071747, rs17878790 sites; CAT gene rs7943316, rs1049982, rs769217 sites; EPHX1 gene rs2234922, rs41266231, rs1051740 sites SNPs and the risk of childhood asthma in Fuzhou, aiming to further elucidate the pathogenesis of asthma and provide new insights and targets for the diagnosis and treatment of bronchial asthma.

## 2 Materials and methods

### 2.1 Study population

This study enrolled children with newly diagnosed bronchial asthma treated at the Department of Pediatrics, Fujian Provincial Hospital, from 2022 to 2023. Inclusion criteria for bronchial asthma were based on the diagnostic criteria outlined in the “2016 Guideline for Diagnosis and Prevention of Childhood Asthma.” Exclusion criteria were as follows: (1) bronchiectasis; (2) primary ciliary dyskinesia; (3) congenital heart disease; (4) bronchopulmonary dysplasia; (5) not being a new diagnosis; (6) lack of family cooperation. Additionally, 50 healthy children undergoing routine health examinations during the same period were enrolled as the control group.

Environmental exposure data, including passive smoking (at least one household memberexposure to indoor tobacco smoke) and allergy history (confirmed by IgE serology positive or physician-diagnosed atopy), along with familial asthma history (in first-degree relatives), were collected using standardized questionnaires.

### 2.2 Research methods

#### 2.2.1 Specimen collection and DNA extraction

Peripheral blood samples of 3 ml were collected from the study subjects in EDTA tubes and stored at −80 °C. DNA extraction from the collected samples was performed using the blood genomic DNA extraction kit from TianGen Biotech Co., Ltd. The extracted DNA samples were subjected to quality control.

#### 2.2.2 Library construction and sequencing

The extracted genomic DNA from the samples was randomly fragmented using the Covaris ultrasonic disruptor to obtain fragments ranging from 180 to 250 base pairs. End repair and A-tailing were performed on the fragmented DNA. Sequencing adapters were ligated to prepare DNA libraries. The library construction was carried out using the Agilent SureSelect Human All Exon V6 exon capture kit. The libraries with specific indexes were hybridized with biotinylated probes. The targeted capture of exon sequences of genes was performed using streptavidin-coated magnetic beads. PCR amplification was conducted to linearly amplify the libraries, completing the library preparation process. The Illumina Nova Seq 6,000 sequencing platform was used for next-generation sequencing.

#### 2.2.3 Construction of genetic models

In molecular genetic epidemiology, genetic model analysis is commonly used to assess the association between genotypes composed of different alleles at various loci and diseases. Currently used models include the heterozygous model, homozygous model, dominant model, recessive model, and allelic model. In this study, genetic models were constructed for 10 SNPs in 100 children sequenced and enrolled in the cohort, aiming to analyze the association between GSTP1, CAT, HMOX1, EPHX1 gene SNPs and the risk of childhood asthma in the Fuzhou region.

Based on the age of asthma onset, participants were stratified into two groups: those greater than 6 years old and less than 6 years old. The same genetic models were employed for stratified analyses, with adjustment for potential confounders including sex, familial asthma history, atopy status, and environmental tobacco smoke exposure.

#### 2.2.4 Linkage disequilibrium analysis and haplotype analysis

The genetic loci included in the study were analyzed for linkage disequilibrium (LD) and haplotype using SHEsis. LD analysis results were represented in the form of a heatmap, with standardized LD coefficient *D*^,^ or *r*^2^ values ≥0.8 indicating strong LD, and values ≤0.1 indicating no LD.

#### 2.2.5 Interaction analysis

Multifactor dimensionality reduction (MDR) was employed to analyze interactions among antioxidant enzyme gene loci, investigating the correlation between multi-locus interactions and the risk of childhood asthma in the Fuzhou region. The best model was selected based on tests of balance accuracy and cross-validation consistency.

### 2.3 Statistical methods

Statistical analyses were performed using SPSS 24 software. Parametric data following a normal distribution were analyzed using *t*-tests, while categorical data were analyzed using chi-square tests. Non-parametric Mann–Whitney *U* tests were used for non-normally distributed data. Both the asthma group and the control group underwent Hardy–Weinberg equilibrium testing. Loci that conformed to Hardy–Weinberg equilibrium (*P* > 0.05) were considered representative of the population and included in subsequent analyses. Binary logistic regression analysis was employed to investigate the association between selected SNP loci and asthma. Sample size calculation and statistical power analysis were conducted using G-Power 3.1.9.7. The estimation parameters were set as follows: odds ratio (OR) = 2.0, two-tailed *α* = 0.05, power (1 −* β*) = 80%, and medium effect size (Cohen's *w* = 0.25).

## 3 Results

### 3.1 Analysis of characteristics of asthma and control groups

The median age (interquartile range) was comparable between the asthma group (7 [5–10] years) and control group (6 [3–11] years; *P* = 0.42). However, significant differences were observed in gender distribution, with male predominance in the asthma group (37 males vs. 13 females) compared to controls (26 males vs. 24 females; *P* = 0.023). The asthma group demonstrated significantly higher prevalence rates of: Familial asthma history (*P* = 0.003), Passive smoking (*P* = 0.019), Atopic status (*P* = 0.002) (Table 1).

**Table 1:** Comparisons of baseline characteristics between asthma and control group.

Parameters	Asthma (*n* = 50)	Control (*n* = 50)	*P*
Age (years)	7 (5,10)	6 (3,11)	0.42
Male sex, *n* (%)	37 (74%)	26 (52%)	***0***.***023***
Family history of asthma, *n* (%)	22 (44%)	8 (16%)	***0***.***003***
Passive smoke exposure, *n* (%)	30 (60%)	18 (36%)	***0***.***019***
Allergy history, *n* (%)	35 (70%)	20 (40%)	***0***.***002***

Italicized bold numbers indicate *P* < 0.05.

Age comparison was conducted using the Mann–Whitney *U* test, categorical variables comparison was conducted using the chi-square test.

### 3.2 Hardy–Weinberg equilibrium test

The test results showed *P* > 0.05 for all comparisons, indicating that there were no statistically significant differences between the theoretical and observed frequencies of wild-type, heterozygous mutant, and homozygous mutant genotypes for the 10 SNPs in GSTP1, HMOX1, CAT, and EPHX1 genes between the two sample groups. This suggests that the samples selected for this study were derived from populations in Hardy–Weinberg equilibrium, indicating good representativeness of the samples.

### 3.3 Analysis of SNPs in GSTP1, HMOX1, CAT, and EPHX1 genes and their correlation with asthma

Association analysis results showed that SNPs at GSTP1 gene rs1695, rs4891, HMOX1 gene rs17878790, CAT gene rs7943316, and rs769217 loci are associated with the risk of childhood asthma in Fuzhou region. Genetic model distributions of HMOX1 gene rs2071747, CAT gene rs1049982, EPHX1 gene rs41266231, rs1051740, rs2234922 did not significantly differ between the asthma group and the control group.

The AG and GG genotypes, the G allele, at GSTP1 gene rs1695 locus increase the risk of asthma. The TC and CC genotypes, the C allele, at rs4891 locus increase the risk of asthma. The GA and AA genotypes, the A allele, at HMOX1 gene rs17878790 locus increase the risk of asthma. The TT genotype and T allele at CAT gene rs7943316 locus increase the risk of asthma. The TT genotype and T allele at rs769217 locus decrease the risk of asthma (Table 2).

**Table 2:** Binary logistic regression analysis of associations between SNPs of GSTP1, HMOX1, CAT, EPHX1 gene and asthma.

Genetic model	Genotype	Asthma	Control	OR (95%CI)	*P*
GSTP1 rs1695 A>G					
Wild-type	AA	21 (42%)	33 (66%)		
Heterozygous	AG	26 (52%)	16 (32%)	2.45 (1.08–5.55)	***0***.***032***
Homozygous	GG	3 (6%)	1 (2%)	4.62 (0.42–50.78)	0.210
Dominant	AA	21 (42%)	33 (66%)		
	AG + GG	29 (58%)	17 (34%)	2.58 (1.09–6.11)	***0***.***031***
Recessive	AA + AG	47 (94%)	49 (98%)		
	GG	3 (6%)	1 (2%)	3.15 (0.29–34.20)	0.342
Allele	A	68 (68%)	82 (82%)		
	G	32 (32%)	18 (18%)	1.95 (1.00–3.82)	***0***.***049***
Genetic model	Genotype	Asthma	Control	OR (95%CI)	*P*
GSTP1 rs4891 T>C					
Wild-type	TT	21 (42%)	32 (64%)		
Heterozygous	TC	26 (52%)	17 (34%)	2.30 (1.02–5.20)	***0***.***045***
Homozygous	CC	3 (6%)	1 (2%)	4.50 (0.41–49.52)	0.218
Dominant	TT	21 (42%)	32 (64%)		
	TC + CC	29 (58%)	18 (36%)	2.37 (1.02–5.53)	***0***.***046***
Recessive	TT + TC	47 (94%)	49 (98%)		
	CC	3 (6%)	1 (2%)	3.02 (0.28–32.85)	0.361
Allele	T	68 (68%)	81 (81%)		
	C	32 (32%)	19 (19%)	1.90 (1.01–3.58)	***0***.***047***
HMOX1 rs2071747 G>C					
Wild-type	GG	46 (92%)	48 (96%)		
Heterozygous	GC	4 (8%)	2 (4%)	1.32 (0.22–7.95)	0.762
Homozygous	CC	0 (0%)	0 (0%)	—	—
Dominant	GG	46 (92%)	48 (96%)		
	GC + CC	4 (8%)	2 (4%)	1.32 (0.22–7.95)	0.762
Recessive	GG + GC	50 (100%)	50 (100%)		
	CC	0 (0%)	0 (0%)	—	—
Allele	G	96 (96%)	98 (98%)		
	C	4 (4%)	2 (2%)	1.30 (0.22–7.68)	0.775
HMOX1 rs17878790 G>A					
Wild-type	GG	40 (80%)	49 (98%)		
Heterozygous	GA	9 (18%)	1 (2%)	8.80 (1.05–73.82)	***0***.***045***
Homozygous	AA	1 (2%)	0 (0%)	—	—
Dominant	GG	40 (80%)	49 (98%)		
	GA + AA	10 (20%)	1 (2%)	9.62 (1.15–80.47)	***0***.***037***
Recessive	GG + GA	49 (98%)	50 (100%)		
	AA	1 (2%)	0 (0%)	—	—
Allele	G	89 (89%)	99 (99%)		
	A	11 (11%)	1 (1%)	11.02 (1.33–91.42)	***0***.***026***
CAT rs7943316 A>T					
Wild-type	AA	20 (40%)	28 (56%)		
Heterozygous	AT	19 (38%)	18 (36%)	1.60 (0.64–4.00)	0.320
Homozygous	TT	11 (22%)	4 (8%)	3.78 (1.02–14.02)	***0***.***046***
Dominant	AA	20 (40%)	28 (56%)		
	AT + TT	30 (60%)	22 (44%)	2.20 (0.93–5.20)	0.073
Recessive	AA + AT	39 (78%)	46 (92%)		
	TT	11 (22%)	4 (8%)	3.85 (1.05–14.16)	***0***.***042***
Allele	A	59 (59%)	74 (74%)		
	T	41 (41%)	26 (26%)	1.92 (1.05–3.52)	***0***.***035***
Genetic model	Genotype	Asthma	Control	OR (95%CI)	*P*
CAT rs1049982 T>C					
Wild-type	TT	23 (46%)	28 (56%)		
Heterozygous	TC	19 (38%)	18 (36%)	1.30 (0.55–3.10)	0.550
Homozygous	CC	8 (16%)	4 (8%)	2.15 (0.57–8.15)	0.256
Dominant	TT	23 (46%)	28 (56%)		
	TC + CC	27 (54%)	22 (44%)	1.52 (0.67–3.45)	0.320
Recessive	TT + TC	42 (84%)	46 (92%)		
	CC	8 (16%)	4 (8%)	2.15 (0.57–8.15)	0.256
Allele	T	65 (65%)	74 (74%)		
	C	35 (35%)	26 (26%)	1.48 (0.82–2.68)	0.195
CAT rs769217 C>T					
Wild-type	CC	18 (36%)	13 (26%)		
Heterozygous	CT	29 (58%)	23 (46%)	0.85 (0.34–2.15)	0.730
Homozygous	TT	3 (6%)	14 (28%)	0.14 (0.03–0.61)	***0***.***009***
Dominant	CC	18 (36%)	13 (26%)		
	CT + TT	32 (64%)	37 (74%)	0.60 (0.25–1.45)	0.260
Recessive	CC + CT	47 (94%)	36 (72%)		
	TT	3 (6%)	14 (28%)	0.15 (0.03–0.63)	***0***.***010***
Allele	C	65 (65%)	49 (49%)		
	T	35 (35%)	51 (51%)	0.48 (0.26–0.89)	***0***.***020***
EPHX1 rs41266231 G>A					
Wild-type	GG	35 (70%)	31 (62%)		
Heterozygous	GA	13 (26%)	18 (36%)	0.62 (0.26–1.48)	0.285
Homozygous	AA	2 (4%)	1 (2%)	2.45 (0.19–31.58)	0.490
Dominant	GG	35 (70%)	31 (62%)		
	GA + AA	15 (30%)	19 (38%)	0.65 (0.27–1.55)	0.335
Recessive	GG + GA	48 (96%)	49 (98%)		
	AA	2 (4%)	1 (2%)	2.85 (0.22–36.90)	0.430
Allele	G	83 (83%)	80 (80%)		
	A	17 (17%)	20 (20%)	0.80 (0.38–1.68)	0.560
EPHX1 rs1051740 T>C					
Wild-type	TT	16 (32%)	22 (44%)		
Heterozygous	TC	25 (50%)	19 (38%)	1.65 (0.70–3.90)	0.252
Homozygous	CC	9 (18%)	9 (18%)	1.80 (0.55–5.90)	0.330
Dominant	TT	16 (32%)	22 (44%)		
	TC + CC	34 (68%)	28 (56%)	1.68 (0.71–3.99)	0.235
Recessive	TT + TC	41 (82%)	41 (82%)		
	CC	9 (18%)	9 (18%)	1.38 (0.45–4.25)	0.580
Allele	T	57 (57%)	63 (63%)		
	C	43 (43%)	37 (37%)	1.25 (0.72–2.18)	0.430
Genetic model	Genotype	Asthma	Control	OR (95%CI)	*P*
EPHX1 rs2234922 A>G					
Wild-type	AA	42 (84%)	41 (82%)		
Heterozygous	AG	8 (16%)	9 (18%)	0.70 (0.24–2.05)	0.520
Homozygous	GG	0 (0%)	0 (0%)	—	—
Dominant	AA	42 (84%)	41 (82%)		
	AG + GG	8 (16%)	9 (18%)	0.72 (0.24–2.15)	0.558
Recessive	AA + AG	50 (100%)	50 (100%)		
	GG	0 (0%)	0 (0%)	—	—
Allele	A	92 (92%)	91 (91%)		
	G	8 (8%)	9 (9%)	0.85 (0.31–2.35)	0.760

Italicized bold numbers indicate *P* < 0.05. All models controlled for age (continuous), sex (male/female), family history (yes/no), passive smoking (yes/no), allergy history (yes/no).

### 3.4 Age-stratified analysis of associations between SNPs of GSTP1, HMOX1, CAT, EPHX1 gene and asthma

The age-stratified analysis showed that the asthma risk effects of GSTP1 rs1695, rs4891, CAT rs7943316, and HMOX1 rs17878790 were more significant in the >6-year-old group. The protective effect of the CAT rs769217 TT genotype was significant in all groups. No age-specific associations were observed for all loci of the EPHX1 gene (rs2234922, rs41266231, rs1051740), HMOX1 rs2071747, or CAT rs1049982 (Table 3).

**Table 3:** Age-stratified analysis of associations between SNPs of GSTP1, HMOX1, CAT, EPHX1 gene and asthma.

Genetic model	≤6 years (*n* = 52)		>6 years (*n* = 48)	
GSTP1 rs1695 A>G	Genotype (Asthma/Control)	OR (95% CI) [*P*-value]	Genotype (Asthma/Control)	OR (95% CI) [*P*-value]
Heterozygous	AG:14/11	***2.05 (1.02–4.12) [0.044]***	AG:12/5	***2.80 (1.25–6.30) [0.012]***
Homozygous	AA:12/22 GG:1/0	4.10 (0.35–48.2) [0.270]	AA:9/11 GG:2/1	***5.25 (1.15–24.0) [0.032]***
Dominant	AA:12/22 AG + GG:15/11	2.10 (0.85–5.18) [0.108]	AA:9/11 AG + GG:9/5	***3.05 (1.12–8.30) [0.029]***
Recessive	AA + AG:26/33 GG:1/0	3.15 (0.28–35.7) [0.352]	AA + AG:21/16 GG:2/1	***4.80 (1.30–17.7) [0.018]***
Allele	A:38/44 G:14/8	1.75 (0.82–3.72) [0.148]	A:30/38 G:18/10	***2.20 (1.10–4.40) [0.026]***
GSTP1 rs4891T>C				
Heterozygous	TC:14/11	***2.15 (1.05–4.40) [0.036]***	TC:12/5	***2.60 (1.20–5.65) [0.016]***
Homozygous	TT:12/21 CC:1/0	4.00 (0.30–53.5) [0.290]	TT:9/11 CC:2/1	***5.15 (1.10–24.2) [0.037]***
Dominant	TT:12/21 TC + CC:15/11	2.15 (0.88–5.25) [0.092]	TT:9/11 TC + CC:9/5	***2.80 (1.05–7.45) [0.040]***
Recessive	TT + TC:26/32 CC:1/0	3.05 (0.25–37.2) [0.380]	TT + TC:21/16 CC:2/1	***4.60 (1.25–16.9) [0.022]***
Allele	T:38/43 C:14/9	1.80 (0.85–3.81) [0.124]	T:30/38 C:18/10	***2.05 (1.02–4.12) [0.044]***
HMOX1 rs2071747 G>C				
Heterozygous	GC:4/2 CC:0/0	1.32 (0.22–7.95) [0.762]	GC:0/0 CC:0/0	—
Homozygous	GG:23/24 CC:0/0	—	GG:25/25 CC:0/0	—
Dominant	GG:23/24 GC + CC:4/2	1.32 (0.22–7.95) [0.762]	GG:25/25 GC + CC:0/0	—
Recessive	GG + GC:27/26 CC:0/0	—	GG + GC:25/25 CC:0/0	—
Allele	G:46/48 C:4/2	1.30 (0.22–7.68) [0.775]	G:50/50 C:0/0	—
HMOX1 rs17878790 G>A				
Heterozygous	GA:6/1 AA:1/0	***8.80 (1.05–73.8) [0.045]***	GA:10/0 AA:0/0	—
Homozygous	GG:20/25 AA:1/0	—	GG:20/25 AA:0/0	—
Dominant	GG:20/25 GA + AA:7/1	***9.62 (1.15–80.5) [0.037]***	GG:20/25 GA + AA:4/0	***18.0 (2.10–154) [0.008]***
Recessive	GG + GA:26/26 AA:1/0	—	GG + GA:24/25 AA:0/0	—
Allele	G:46/49 A:6/1	***7.80 (1.05–58.0) [0.045]***	G:40/50 A:10/0	***∞ (3.12***—***∞) [0.001]***
CAT rs7943316 A>T				
Heterozygous	AT:13/11	1.60 (0.64–4.00) [0.320]	AT:11/9	1.85 (0.70–4.90) [0.210]
Homozygous	AA:12/22 TT:3/1	***3.20 (1.05–9.78) [0.041]***	AA:9/11 TT:4/1	***4.85 (1.55–15.2) [0.006]***
Dominant	AA:12/22 AT + TT:15/11	2.20 (0.93–5.20) [0.073]	AA:9/11 AT + TT:9/5	***2.80 (1.15–6.85) [0.023]***
Recessive	AA + AT:25/33 TT:3/1	***3.85 (1.05–14.2) [0.042]***	AA + AT:20/20 TT:4/1	***5.20 (1.55–17.5) [0.007]***
Allele	A:37/44 T:15/8	***1.85 (1.02–3.35) [0.042]***	A:29/38 T:19/10	***2.40 (1.25–4.60) [0.008]***
CAT rs1049982 T>C				
Heterozygous	TC:13/11	1.30 (0.55–3.10) [0.550]	TC:11/9	1.45 (0.60–3.52) [0.410]
Homozygous	TT:12/22 CC:3/1	2.15 (0.57–8.15) [0.256]	TT:9/11 CC:4/1	2.30 (0.60–8.85) [0.230]
Dominant	TT:12/22 TC + CC:15/11	1.52 (0.67–3.45) [0.320]	TT:9/11 TC + CC:9/5	1.65 (0.72–3.80) [0.235]
Recessive	TT + TC:25/33 CC:3/1	2.15 (0.57–8.15) [0.256]	TT + TC:20/20 CC:4/1	2.30 (0.60–8.85) [0.230]
Allele	T:37/44 C:15/8	1.48 (0.82–2.68) [0.195]	T:29/38 C:19/10	1.55 (0.85–2.82) [0.150]
CAT rs769217 C>T				
Heterozygous	CT:13/13	0.85 (0.34–2.15) [0.730]	CT:11/8	0.80 (0.32–2.00) [0.630]
Homozygous	CC:12/13 TT:1/8	***0.12 (0.01–0.99) [0.049]***	CC:9/10 TT:1/6	***0.10 (0.01–0.85) [0.035]***
Dominant	CC:12/13 CT + TT:13/13	0.60 (0.25–1.45) [0.260]	CC:9/10 CT + TT:9/6	0.55 (0.22–1.35) [0.190]
Recessive	CC + CT:25/26 TT:1/8	***0.15 (0.03–0.63) [0.010]***	CC + CT:20/18 TT:1/6	***0.12 (0.02–0.60) [0.010]***
Allele	C:37/26 T:15/26	***0.45 (0.25–0.81) [0.007]***	C:29/24 T:19/24	***0.40 (0.22–0.75) [0.004]***
EPHX1 rs41266231 G>A				
Heterozygous	GA:9/10 AA:1/2	0.62 (0.26–1.48) [0.285]	GA:9/10 AA:1/1	0.65 (0.28–1.52) [0.320]
Homozygous	GG:17/15 AA:1/2	2.45 (0.19–31.6) [0.490]	GG:16/15 AA:1/1	2.60 (0.20–33.8) [0.470]
Dominant	GG:17/15 GA + AA:9/10	0.65 (0.27–1.55) [0.335]	GG:16/15 GA + AA:9/10	0.68 (0.29–1.60) [0.380]
Recessive	GG + GA:26/25 AA:1/2	2.85 (0.22–36.9) [0.430]	GG + GA:25/25 AA:1/1	3.00 (0.23–39.2) [0.410]
Allele	G:43/42 A:9/10	0.80 (0.38–1.68) [0.560]	G:41/40 A:9/10	0.85 (0.40–1.80) [0.670]
EPHX1 rs1051740 T>C				
Heterozygous	TC:13/11 CC:5/4	1.65 (0.70–3.90) [0.252]	TC:12/10 CC:4/3	1.75 (0.75–4.10) [0.190]
Homozygous	TT:8/11 CC:5/4	1.80 (0.55–5.90) [0.330]	TT:8/10 CC:4/3	1.95 (0.60–6.35) [0.260]
Dominant	TT:8/11 TC + CC:14/11	1.68 (0.71–3.99) [0.235]	TT:8/10 TC + CC:12/10	1.80 (0.77–4.20) [0.170]
Recessive	TT + TC:21/22 CC:5/4	1.38 (0.45–4.25) [0.580]	TT + TC:20/20 CC:4/3	1.45 (0.48–4.40) [0.510]
Allele	T:29/33 C:23/19	1.25 (0.72–2.18) [0.430]	T:28/30 C:20/18	1.30 (0.75–2.25) [0.350]
EPHX1 rs2234922 A>G				
Heterozygous	AG:6/5 GG:0/0	0.70 (0.24–2.05) [0.520]	AG:6/5 GG:0/0	0.75 (0.26–2.20) [0.600]
Homozygous	AA:22/21 GG:0/0	—	AA:20/20 GG:0/0	—
Dominant	AA:22/21 AG + GG:6/5	0.72 (0.24–2.15) [0.558]	AA:20/20 AG + GG:6/5	0.78 (0.27–2.25) [0.642]
Recessive	AA + AG:28/26 GG:0/0	—	AA + AG:26/25 GG:0/0	—
Allele	A:46/47 G:6/5	0.85 (0.31–2.35) [0.760]	A:44/45 G:6/5	0.90 (0.33–2.45) [0.830]

Italicized bold numbers indicate *P* < 0.05. All models controlled for sex (male/female), family history (yes/no), passive smoking (yes/no), allergy history (yes/no).

### 3.5 Power analysis

The minimum required sample size was 98 subjects (49 per group). Our final cohort (50 asthma/50 controls, *n* = 100) achieved 82%–92% *post-hoc* power for OR ≥ 2.0. Power calculations demonstrated variable detection capacity across effect size ranges: (1) high power (>80%) for clinically significant effects (OR ≥ 2.0 or ≤0.5). (2) moderate power (70%) for intermediate effects (1.5 ≤ OR < 2.0). (3) limited power (35%) for small effects (OR < 1.5). (4) suboptimal power (55%) for rare variants (Table 4).

**Table 4:** Power summary.

Effect magnitude	OR range	Mean PowerTesting bal	Conclusion
High power	≥2.0 or ≤0.5	85%	Adequate
Moderate power	1.5–2.0	70%	Marginal
Small	<1.5	35%	Inadequate
Suboptimal power	MAF < 5%	55%	Inadequate

### 3.6 Linkage disequilibrium analysis of GSTP1, HMOX1, CAT, and EPHX1

**Figure 1:**
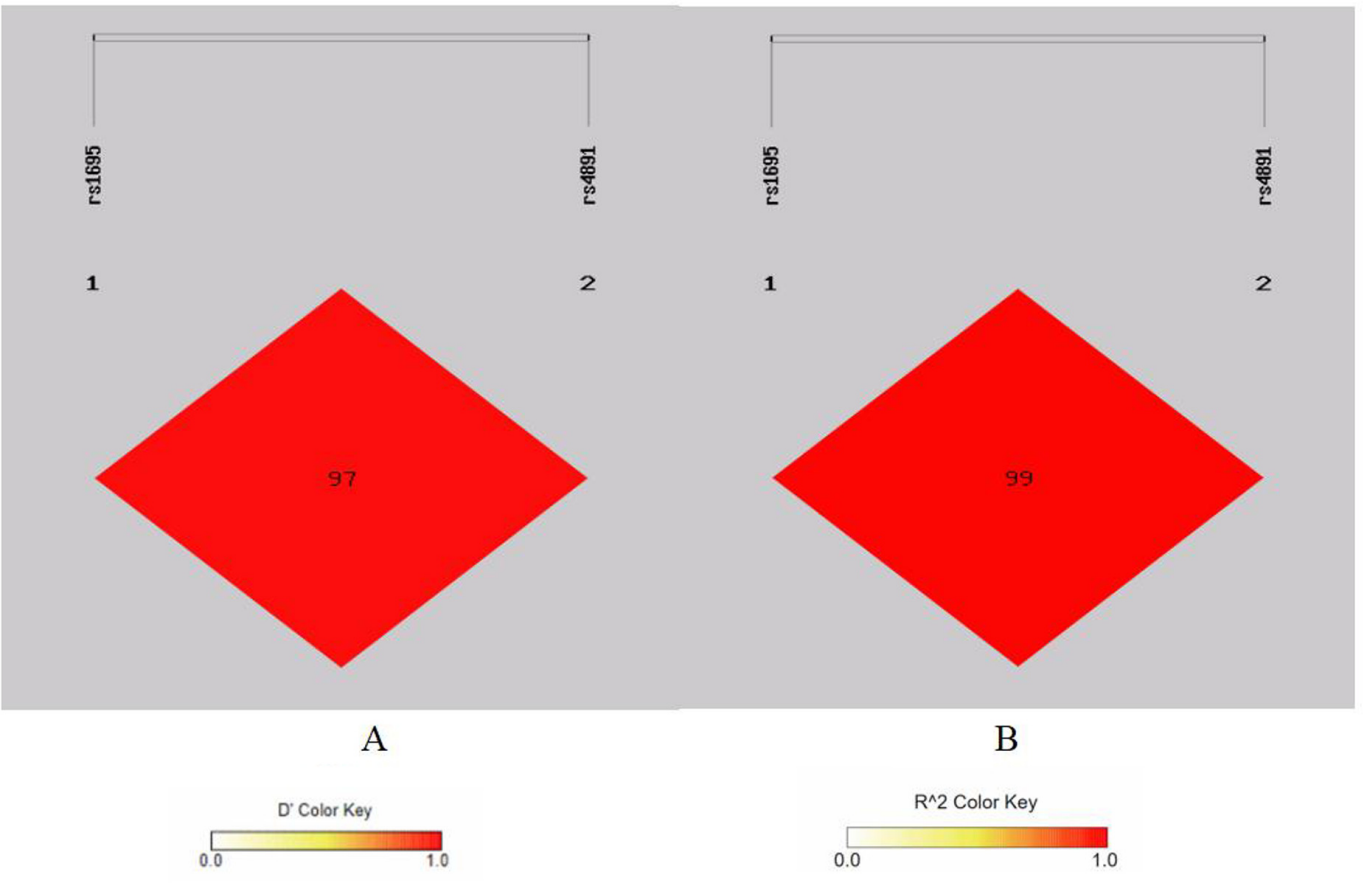
Linkage disequilibrium analysis at rs1695, rs4891 of GSTP 1. **(A)** Means *D*′; **(B)** means *r*^2^.

**Figure 2:**
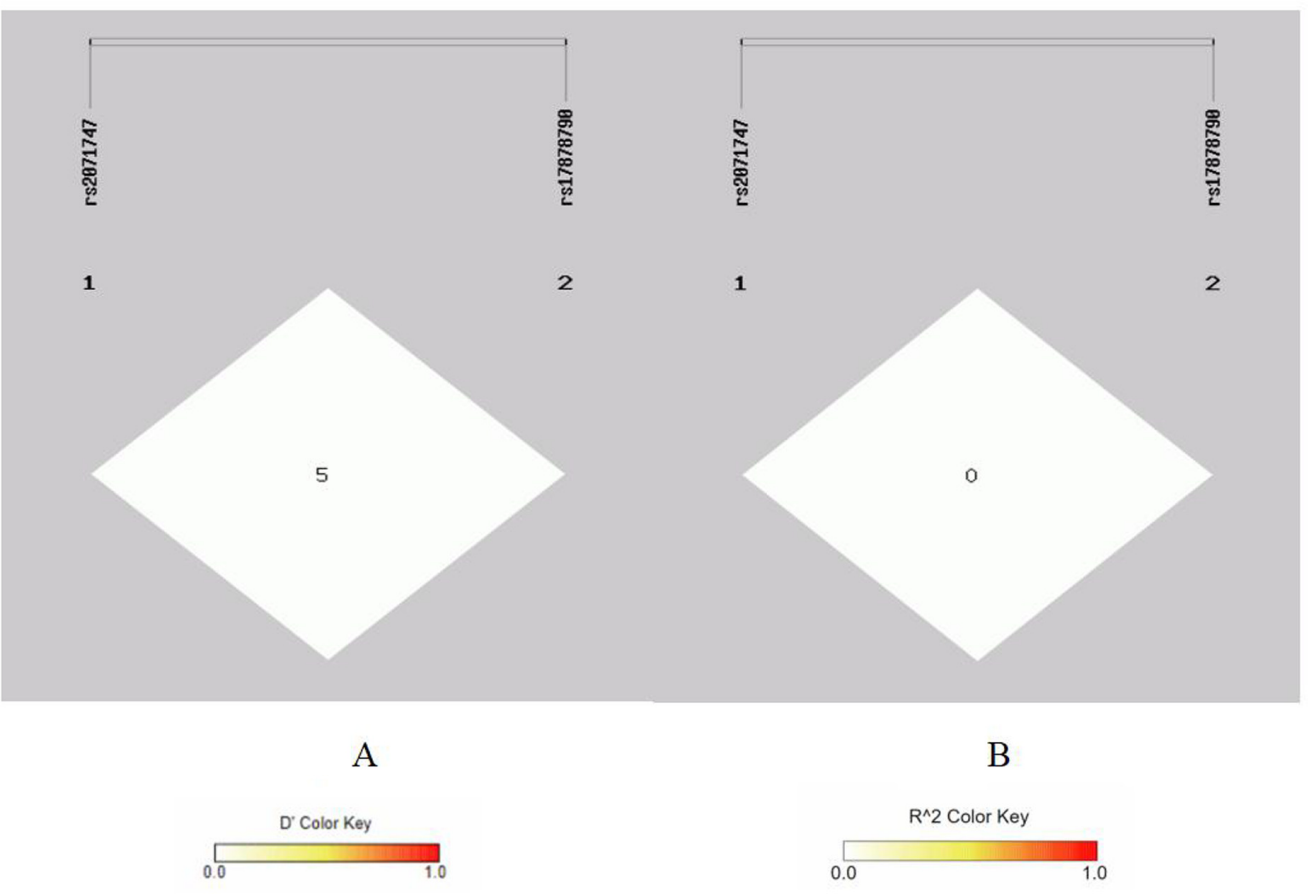
Linkage disequilibrium analysis at rs2071747, rs17878790 of HMOX1. **(A)** Means *D*′; **(B)** means *r*^2^.

**Figure 3:**
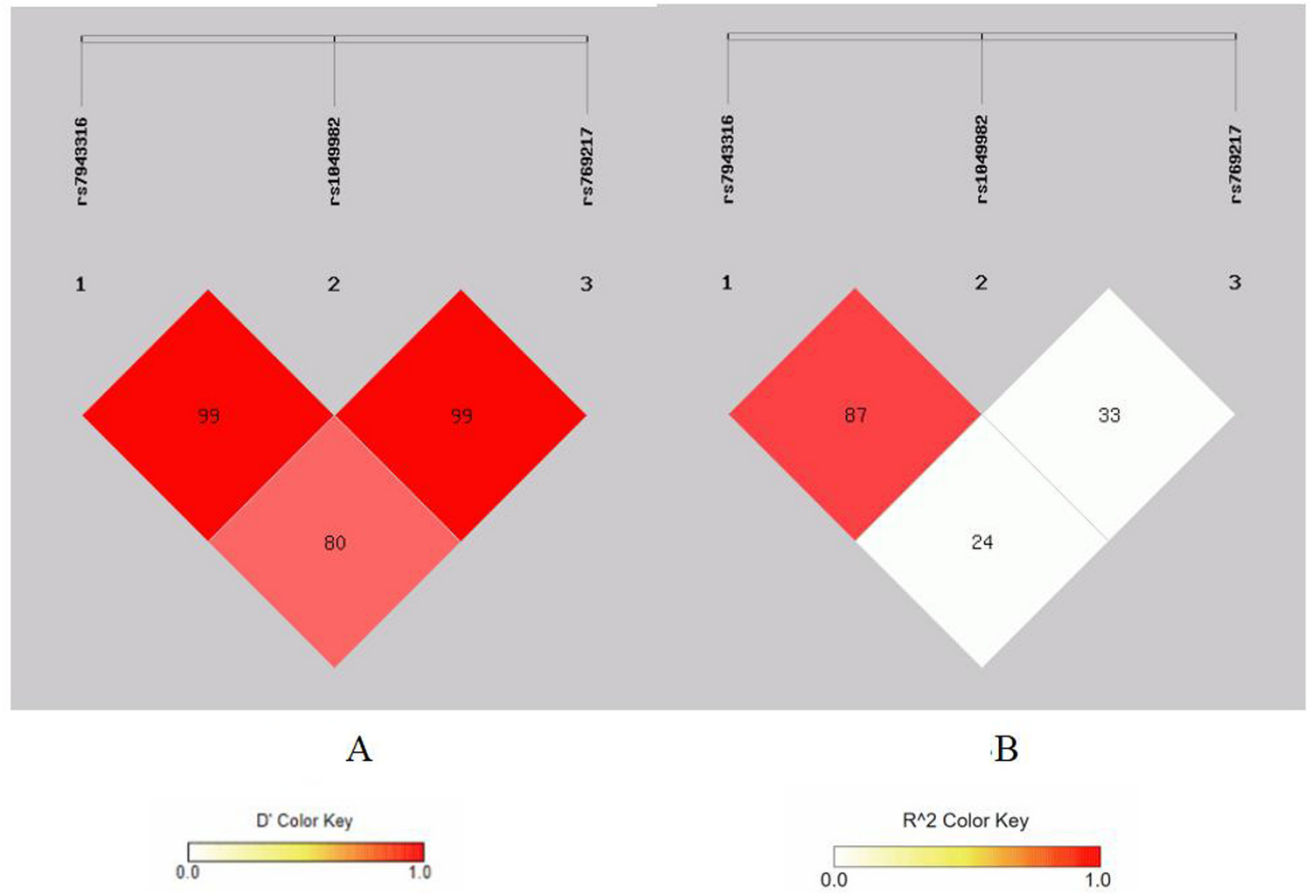
Linkage disequilibrium analysis at rs7943316, rs1049982, rs7692 17 of CAT. **(A)** means *D*′; **(B)** means *r*^2^.

**Figure 4:**
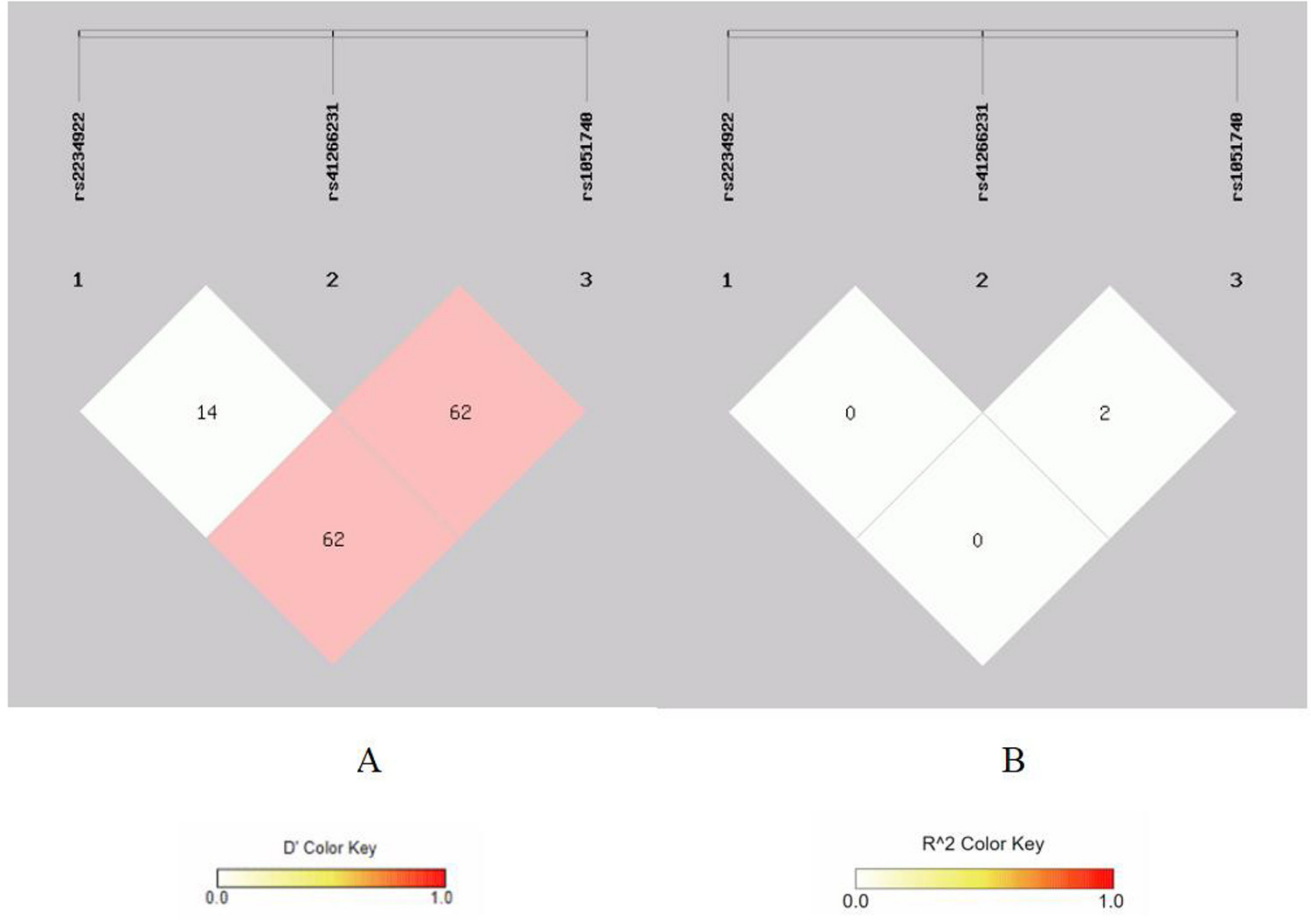
Linkage disequilibrium analysis at rs2234922, rs41266231, rs 1051740 of EPHX1. **(A)** Means *D*′; **(B)** means *r*^2^.

There is strong linkage disequilibrium between rs1695 and rs4891 loci, and three loci (rs7943316, rs1049982, and rs769217) exhibit three types of strong linkage disequilibrium (Figures 1–4).

### 3.7 Haplotype analysis of GSTP1 and CAT

Genotypic GC composed of rs1695 and rs4891 in the GSTP1 gene may represent a risk haplotype for childhood asthma, while genotypic AT may represent a protective haplotype for childhood asthma (Table 5). Haplotype ATT composed of rs7943316, rs1049982, and rs769217 in the CAT gene may potentially represent a protective haplotype for the risk of childhood asthma (Table 6).

**Table 5:** Haplotype analysis of rs1695, rs4891 of GSTP1.

GSTP1 SNPs		Freq		OR (95%CI)	*P*
rs1695	rs4891	Asthma	Control		
A	T	0.68	0.81	0.47 (0.24–0.92)	***0***.***025***
G	C	0.32	0.18	2.12 (1.09–4.10)	***0***.***025***

Italicized bold numbers indicate *P* < 0.05.

**Table 6:** Haplotype analysis of rs7943316, rs1049982, rs769217 of CAT.

CAT SNPs			Freq		OR (95%CI)	*P*
rs7943316	rs1049982	rs769217	Asthma	Control		
A	T	C	0.28	0.23	1.34 (0.70–2.54)	0.370
A	T	T	0.31	0.51	0.45 (0.25–0.79)	***0***.***006***
T	C	C	0.35	0.26	1.58 (0.86–2.91)	0.139

Italicized bold numbers indicate *P* < 0.05.

### 3.8 Interaction analysis of SNP at GSTP1, HMOX1, CAT, and EPHX1

#### 3.8.1 The best model

Analysis of SNP Interactions among GSTP1, HMOX1, CAT, and EPHX1 Loci Using MDR 3.0.2 Software. The results showed three models among 10 SNPs from four antioxidant enzyme genes: rs1695; rs1695, rs7943316; rs1695, rs7943316, rs41266231. The two-locus model consisting of rs1695 and rs7943316 exhibited the highest testing balance accuracy and cross-validation consistency (testing balance accuracy = 0.68, cross-validation consistency = 10/10). Additionally, this two-locus model had a *P* value < 0.05, indicating that rs1695 and rs7943316 constitute the optimal model (Table 7).

**Table 7:** Interaction analysis of SNPs of GSTP1, HMOX1, CAT, EPHX1.

Model	Training bal	Testing bal	CVC	*P*
rs1695	0.6211	0.6	9/10	***0***.***0161***
rs1695, rs7943316	0.69	0.68	10/10	***0***.***0001***
rs1695, rs7943316, rs41266231	0.7522	0.66	9/10	***0***.***0001***

Italicized bold numbers indicate *P* < 0.05.

#### 3.8.2 Analysis of SNP interactions among rs1695, rs7943316, and rs41266231

**Figure 5:**
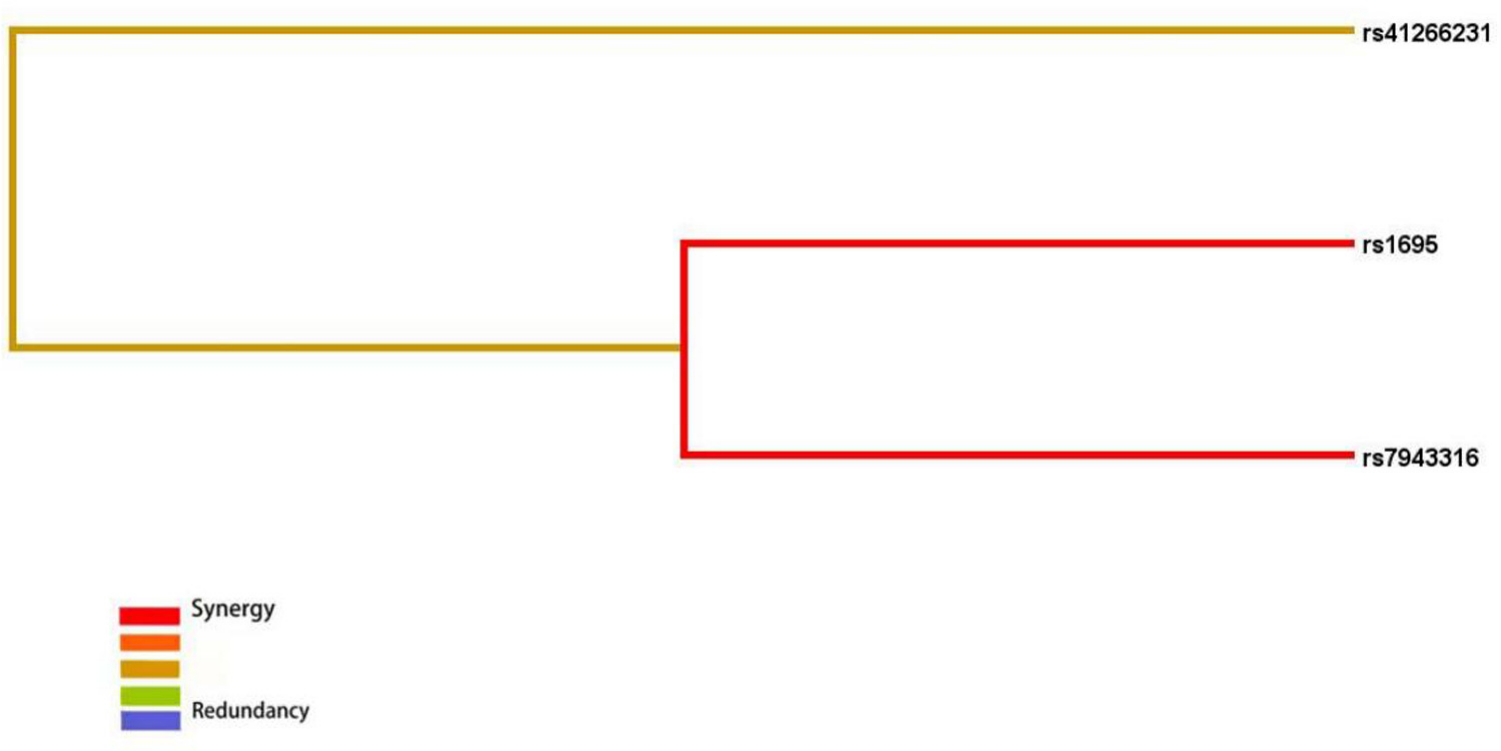
Interaction tree diagram of rs1695, rs7943316, rs41266231. Red means strong_synergy, Yellow means moderate synergy, Blue means redundancy.

All three models have *P* values < 0.05, indicating their significance. This suggests potential interactions among rs1695, rs7943316, and rs41266231. A dendrogram was used to illustrate the types and strengths of these interactions. Results indicate a strong synergistic interaction between rs1695 and rs7943316, a moderately strong synergistic interaction between rs41266231 and rs1695/rs7943316, and suggest that the synergistic interactions among these three loci may increase the risk of childhood asthma in the Fuzhou region (Figure 5).

## 4 Discussion

Asthma is a common chronic heterogeneous disease in children. Currently, there is extensive research on asthma susceptibility genes, confirming associations with genes such as interleukin-4 (IL-4), interleukin-13 (IL-13) (17), *β*2 adrenergic receptor (ADRB2) (18), Zona Pellucida Binding Protein 2 (ZPBP2) (19), and numerous other candidate genes. Previous studies have demonstrated that genes involved in oxidative stress response, including GSTP1, CAT, HMOX1, and EPHX1, participate in the pathogenesis of asthma. These four antioxidant enzyme gene SNPs may affect protein enzyme expression and consequently influence the risk of asthma in children from Fuzhou, China.

GSTP1, located on the long arm of human chromosome 11 in the q13 region, spans 2.8 kb and encodes 210 amino acids distributed across 7 exons and 6 introns. Widely expressed in human airways, GSTP1 functions primarily by catalyzing the binding of numerous hydrophobic and electrophilic compounds to reduced glutathione, thereby inhibiting cellular reactive oxygen species production. Previous studies have linked specific nucleotide sites of this gene to asthma susceptibility. In a case-control study conducted among a Turkish population, individuals homozygous for the rs1695 mutant genotype were found to have a 3.55-fold increased risk of developing asthma in adulthood compared to those with the wild-type genotype (20). In a study by Yu-Fen Li, it was reported that the mutation at rs1695 locus of GSTP1 gene increases the risk of childhood asthma and wheezing, and may exacerbate adverse reactions to tobacco exposure in children (21). Another study, conducted with a sample from the Spanish population, showed that children with mutations at the rs1695 locus have an increased risk of developing asthma by the age of 6, and this risk correlates positively with the number of mutations at the rs1695 locus (22). In this study, genetic model analysis of the rs1695 locus revealed that the rs1695 A>G variant increased the risk of childhood asthma in Fuzhou under heterozygous, dominant, and allelic models. The pathogenic effects likely arise from compromised GSTP1 enzymatic activity, wherein the rs1695 (Ile105Val) missense mutation induces an isoleucine-to-valine substitution at codon 105, resulting in approximately 30% reduction in catalytic efficiency toward electrophilic substrates. This functional impairment diminishes glutathione conjugation capacity, ultimately leading to defective detoxification of reactive oxygen species and heightened cellular vulnerability to oxidative stress. Previous research on the rs4891 locus of the GSTP1 gene is limited, with only a few studies analyzing its SNPs' association with Chronic Obstructive Pulmonary Disease (COPD) and lung cancer (23, 24), Currently, there are no reports on its relationship with asthma onset. In our study, genetic model analysis of the rs4891 locus showed that the rs4891 T>C variant increased the risk of childhood asthma in Fuzhou under heterozygous, dominant, and allelic models. The observed genotype-phenotype association may be mediated through functional alterations of the encoded protein, whereby the T>C nonsynonymous variant causes an alanine-to-valine substitution at residue 114 (Ala114Val). This amino acid substitution is predicted to perturb substrate binding kinetics, although the precise mechanistic consequences remain to be fully elucidated.

HMOX1, an inducible subtype of heme oxygenase, contains 4 introns and 5 exons in its gene structure. HMOX1 catalyzes the degradation of potent oxidant heme to produce antioxidants, thereby exerting an antioxidative stress effect. Several studies indicate that upregulation of HMOX1 expression alleviates airway inflammation in asthmatic mice (25). Researchers including Jiajia Lv found that HMOX1 protects airway epithelial cells of asthma patients from apoptosis (26). These studies collectively demonstrate the antioxidative stress role of HMOX1 in attenuating airway inflammation in asthma. Currently, there is limited research on the correlation between HMOX1 gene SNP loci and asthma onset. In this study, genetic model analysis of rs17878790 site revealed: in heterozygous and dominant models, rs17878790 G>A increases the susceptibility to childhood asthma in Fuzhou. Despite demonstrating a clinically significant OR, the AA genotype of HMOX1 rs17878790 exhibited insufficient statistical power due to low allele frequency in our cohort, necessitating validation through multicenter studies with expanded sample sizes to achieve adequate power for robust association analysis.

Catalase (CAT) is a hallmark enzyme of peroxisomes, constituting 40% of the total peroxisomal enzymes. The CAT gene is located on 11p13, spanning 35 kb, with 13 exons and 12 introns, encoding 526 amino acids. CAT converts H₂O₂ into harmless substances—water and oxygen—thereby playing roles in scavenging free radicals and protecting cells from damage by superoxide anions. The CAT gene rs769217 SNP locus has been reported to correlate with disease risks such as hepatocellular carcinoma, cirrhosis, and glaucoma. Studies by Liu et al. indicate that carriers of the rs769217 site T allele are at increased risk of hepatocellular carcinoma and cirrhosis (27). Research by Belamkar et al. shows that individuals with the rs769217 CC genotype are at higher risk for primary open-angle glaucoma (28, 29). However, there are currently no reports on the correlation between rs769217 SNP loci and asthma. In this study, genetic model analysis of the rs769217 site indicated: in homozygous and recessive models, children with the TT genotype have a decreased risk of asthma; in allele models, the frequency of the T allele is significantly lower in the asthma group compared to the control group. These results suggest that the T allele of rs769217 is a protective allele against asthma, reducing the risk of childhood asthma. Though its precise molecular mechanisms require further investigation and validation. Previous studies indicate that the CAT gene rs7943316 SNP locus is associated with increased risks of diseases such as vitiligo and hearing loss (30), with rare reports on its correlation with asthma onset. In this study, genetic model analys is of the rs7943316 site revealed: in homozygous, recessive, and allele models, rs7943316 A>T increases the susceptibility to childhood asthma in Fuzhou. This regulatory region variant likely exerts its pathogenic effect through transcriptional modulation of CAT expression. This study did not find a correlation between rs1049982 SNP loci and the risk of childhood asthma in the Fuzhou region.

EPHX1 is a member of the *α*/*β*-hydrolase fold epoxide hydrolase (EH) family, which exerts antioxidative effects through its involvement in the metabolism of polycyclic aromatic hydrocarbons, phthalates, and other organic pollutants (31–33). In this study, genetic model analysis revealed no statistically significant differences in the distribution frequencies of genotypes at EPHX1 gene loci rs2234922, rs41266231, and rs1051740 between the asthma and control groups across five genetic models. This study did not find a correlation between these SNP loci and the risk of childhood asthma in the Fuzhou region. The observed null association may be attributed to several potential explanations: 1. true biological irrelevance of these loci in asthma pathogenesis. 2. limited statistical power (1 − *β* < 0.8) to detect modest genetic effects (OR < 1.5) due to sample size constraints. 3. population-specific or environment-dependent penetrance of EPHX1 variants. Given EPHX1's crucial role in metabolizing polycyclic aromatic hydrocarbons, its genetic variants may demonstrate more pronounced effects on asthma susceptibility in populations with significant airborne pollutant exposure. Subsequent investigations employing expanded cohorts and diverse environmental settings are warranted to elucidate these genotype-environment interactions.

Age-stratified analyses demonstrated differential genetic effects, with GSTP1 rs1695, rs4891, CAT rs7943316, and HMOX1 rs17878790 exhibiting stronger asthma risk associations in >6 years group potentially mediated by prolonged oxidative stress accumulation, developmental changes in immune regulation, and environmental exposures. In contrast, the CAT rs769217 variant maintained consistent protective effects across all age strata, suggesting its fundamental role in constitutive antioxidant defense mechanisms rather than age-modulated pathways.

GSTP1 gene SNPs rs1695 A>G, rs4891 T>C, CAT gene SNP rs7943316 A>T are potential risk factors for childhood asthma in the Fuzhou region. CAT gene SNP rs769217 C>T may be a protective factor against childhood asthma in the same region. Changes in these SNP loci may alter the amino acids encoded by genes, thereby affecting protein activity or expression levels, influencing airway antioxidative defense capabilities, and ultimately affecting the susceptibility to childhood asthma. Further mechanistic studies are warranted.

Our analyses revealed strong linkage disequilibrium between GSTP1 polymorphisms (rs1695 and rs4891) and among CAT variants (rs7943316, rs1049982, and rs769217), suggesting their co-inheritance and potential functional synergy. Haplotype analysis identified the GSTP1 GC combination as a significant risk haplotype, while the AT haplotype demonstrated protective effects. These findings are supported by mechanistic studies showing that the GSTP1 Ile105Val substitution (rs1695) decreases catalytic efficiency by 30%–40%, compromising reactive oxygen species detoxification and promoting airway oxidative stress. The rs4891 variant may potentiate this dysfunction through substrate-binding domain alterations, although its exact structural consequences require further crystallographic characterization.

The identified protective ATT haplotype in the CAT gene likely represents a coordinated modulation of catalase function, where the rs7943316 polymorphism may decrease transcriptional efficiency while the rs769217 variant alters protein stability. Mechanistically, the protective rs769217 T allele could counteract the rs7943316-associated risk by preserving catalase's antioxidant capacity through enhanced structural stability, demonstrating how haplotype-specific interactions may collectively regulate redox homeostasis through complementary functional effects.

MDR analysis indicated potential interaction among rs1695, rs7943316, and rs41266231, with rs1695 and rs7943316 forming the best model. There appears to be strong synergistic interaction between rs1695 and rs7943316, where this combination predicts the risk of childhood asthma more effectively than either locus alone. Although this study did not find a correlation between rs41266231 SNP and asthma risk, this locus may contribute to asthma pathogenesis through interactions with rs1695 and rs7943316. Further research is needed to elucidate the specific mechanisms of interaction among these three loci.

The GSTP1 and CAT risk variants identified in our study are established functional polymorphisms known to impair antioxidant enzyme activity. Previous mechanistic studies have demonstrated that the GSTP1 risk alleles (rs1695 and rs4891) are associated with elevated systemic oxidative damage markers, including malondialdehyde (MDA) and advanced oxidation protein products (AOPP). Although our study did not directly measure oxidative stress biomarkers, several indirect evidences support the biological plausibility of our findings: 1.significantly higher environmental tobacco smoke exposure in asthma cases, environmental tobacco smoke exposure is a potent source of reactive oxygen species. 2. Greater atopy prevalence among asthmatics where allergic inflammation generates substantial oxidative stress. The convergence of genetic susceptibility variants with these pro-oxidant environmental exposures suggests that oxidative stress likely serves as a key pathophysiological pathway mediating childhood asthma development.

This study identified significant associations between childhood asthma susceptibility and multiple SNPs in key antioxidant genes (GSTP1, HMOX1, and CAT), suggesting their potential utility as predictive biomarkers. Specifically, the GSTP1 rs1695 G allele conferred increased asthma risk, whereas the CAT rs769217 T allele exhibited protective effects. These findings highlight opportunities for precision medicine approaches, such as: (1) early environmental risk mitigation for genetically high-risk individuals, (2) targeted therapeutic development modulating antioxidant pathways to restore redox homeostasis in susceptible populations.

Several limitations of this study warrant consideration. First, the sample size constrained statistical power for detecting small genetic effects, increasing susceptibility to type II errors, particularly for rare variants. Second, although major covariates were adjusted for, residual confounding from unmeasured environmental or epigenetic factors cannot be excluded. Third, reliance on observational asthma diagnosis rather than objective spirometry may have reduced phenotypic precision, as lung function measures could provide greater sensitivity for detecting genotype-phenotype associations. Fourth, the hospital-based case-control design introduces potential Berkson's bias, limiting generalizability to population-based samples. Finally, these findings should be interpreted as hypothesis-generating and require replication in larger, ethnically diverse prospective cohorts with standardized phenotyping.

Subsequent investigations should prioritize: (1) mechanistic interrogation of gene-environment interactions using *in vitro* or ex vivo models; (2) clinical validation of these SNPs, predictive utility in asthma risk stratification algorithms, incorporating polygenic risk scores and established biomarkers; 3. integrated analyses coupling genotype data with direct oxidative stress profiling to establish causal links between genetic variants, redox dysregulation, and asthma phenotypes.

## Data Availability

The datasets presented in this study can be found in online repositories. The names of the repository/repositories and accession number(s) can be found below: https://www.ncbi.nlm.nih.gov/, PRJNA1019820.
